# GenESysV: a fast, intuitive and scalable genome exploration open source tool for variants generated from high-throughput sequencing projects

**DOI:** 10.1186/s12859-019-2636-5

**Published:** 2019-01-31

**Authors:** Mohammad Zia, Paul Spurgeon, Adrian Levesque, Thomas Furlani, Jianxin Wang

**Affiliations:** 1 Center for Computational Research, University at Buffalo, Buffalo, NY USA; 2 Buffalo Institute for Genomics and Data Analytics, University at Buffalo, Buffalo, NY USA

**Keywords:** High-throughput sequencing, Genotyping, Genome, VCF, WGS, WES, Variants, Annotation, Mendelian diseases, Complex diseases

## Abstract

**Background:**

High throughput sequencing technologies have been increasingly used in basic genetic research as well as in clinical applications. More and more variants underlying Mendelian and complex diseases are being discovered and documented using these technologies. However, identifying and obtaining a short list of candidate disease-causing variants remains challenging for most of the users after variant calling, especially for people without computational skills.

**Results:**

We developed GenESysV (Genome Exploration System for Variants) as a scalable, intuitive and user-friendly open source tool. It can be used in any high throughput sequencing or genotyping project for storing, managing, prioritizing and efficient retrieval of variants of interest. GenESysV is designed for use by researchers from a wide range of disciplines and computational skills, including wet-lab scientists, clinicians, and bioinformaticians.

**Conclusions:**

GenESysV is the first tool to be able to handle genomic variant dataset ranging in size from a few to thousands of samples and still maintain fast data importation and good query performance. It has a very intuitive graphical user interface and can also be used in studies where secured data access is an important concern. We believe this tool will benefit the human disease research community to speed up discoveries for genetic variants underlying human genetic disorders.

**Electronic supplementary material:**

The online version of this article (10.1186/s12859-019-2636-5) contains supplementary material, which is available to authorized users.

## Background

The advent of high throughput sequencing technologies has greatly accelerated the identification of variants that underlie Mendelian and complex diseases [1–7]. With the cost of sequencing decreasing and sequencing accuracy improving, an increasing number of research laboratories/projects have adopted these technologies to interrogate variants from a few or even hundreds to thousands of human samples in an attempt to identify variants that may underline rare monogenic or common complex diseases. Of the millions of variants typically found in any given individual, most of them likely only contribute to human population diversities. Identifying a subset of variants that are most likely underlying the disease or traits of interest requires field knowledge combined with the use of software tools to facilitate this process. The typical workflow in selecting candidate disease-causing variants starts with variant annotation using tools such as the Ensembl Variant Effect Predictor (VEP) [8] or Annovar [9]. Variants are then subsequently filtered by their minor allele frequencies and other criteria such as the functional consequences to the genes and transcripts they affect, conservation scores [10, 11], predicted pathogenicity scores [12, 13], known associations with disease phenotypes [14–16], etc. After these filtering steps, a short list of candidate disease-causing genes or variants can be produced and reviewed by field experts for downstream validation.

Due to a very large number of variants typically identified from a sequencing project, retrieving variants of interest based on the above criteria generally requires writing custom scripts to process VCF [17] format files, a de facto standard used in reporting genetic variants from high throughput sequencing or genotyping projects. Given the importance of identifying these variants, it is not surprising that a number of software tools have been developed in the past few years. These include commercial packages such as Ingenuity Variant Analysis from QIAGEN (www.qiagen.com/ingenuity), VarSeq (http://goldenhelix.com/products/VarSeq/index.html) and Sequence Miner (https://www.wuxinextcode.com), as well as several open source tools, such as GEMINI [18], BrowseVCF [19], VCF-miner [20], Mendel,MD [21] and BiERapp [22].

During the course of supporting genomics projects engaged by the Buffalo Institute for Genomics and Data Analytics (https://www.buffalo.edu/genomics.html), we surveyed existing open source software in order to find a package that would meet our needs for performance, ease of use, scalability, and controlled access to its users and their proprietary data (Table 1). Unfortunately, many of these open source tools are not designed as comprehensive variant exploration tools and are unable to handle all of the commonly known disease models, neither are they designed for use by multiple researchers who require secure data storage and access. Furthermore, many of the existing tools lack rapid analysis capability for large cohorts consisting of thousands of samples and hundreds of millions of variants.

**Table 1:** Comparison of existing open-source software tools with similar functions

Features	GenESysV	GEMINI	BrowseVCF	VCF-Miner	Mendel,MD	BiERapp
Graphical User^a^ Interface	Yes	No	Yes	Yes	Yes	Yes
Study type	Single cohort complex disease, Case/Control, and Mendelian inheritance	Single cohort complex disease and Mendelian inheritance	Single cohort complex and Mendelian inheritance	Single cohort complex disease and Mendelian inheritance	Mendelian only	Single cohort complex disease, Case/Control, and Mendelian inheritance
Whole genome, exome or target study	All	All	All	All	WES or targeted study	WES or target study
Can handle studies with large numbers of samples	Yes	Yes	No	No	No	No
Database Type	Elasticsearch	Sqlite3	Wormtable & BerkeleyDB	MongoDB	PostgreSQL	SQLite & MongoDB
Flag variants for further filtering	Yes	No	No	No	No	No

^a^Features listed here are not exhaustive

To overcome these limitations, we developed GenESysV - an open source software system with an intuitive user interface. GenESysV can be deployed on a single computer or on a multi-node computer cluster to enable a wide range of researchers with varying computational skills to explore and prioritize variants in both coding and non-coding regions of the human genome. It can scale for studies with thousands of samples, yet still gives satisfactory data loading and querying performance. Below, we describe its design, features and performance benchmarks.

## Implementation

### Design overview

**Fig. 1:**
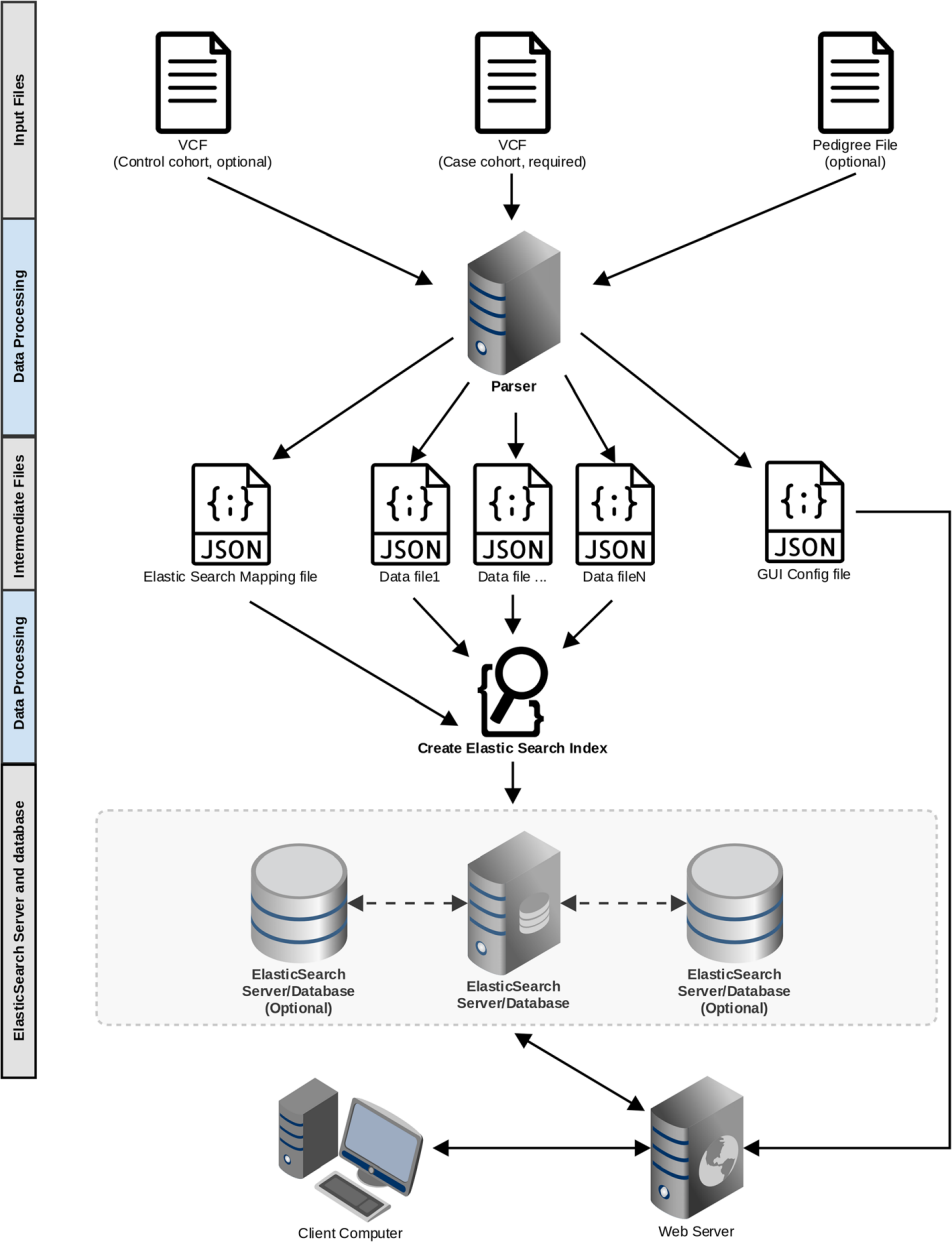
Schematic view of GenESysV design. Input VCF file(s) is parsed into json format files using multiple CPU cores in parallel. An Elasticseach index mapping file, as well as a GUI configure file, are also created during data parsing. The GUI configure file is used to guide the automatic web graphical interface creation in a later stage. Elasticsearch index creation is also done in parallel to further speed up the entire data importation process

Two of the primary goals for developing GenESysV were ease of use for researchers of any computational skill level and good VCF file search performance regardless of the cohort size. Based on these considerations, we designed and implemented GenESysV utilizing the Python Django Web development framework and Elasticsearch, a NoSQL database. The choice of Elasticsearch is based mainly on the sparse nature of genotype data in VCF files. It is optimized both for a workstation environment as well as for a parallel computing environment. To facilitate the development of web applications with an easy-to-use user interface, we chose the Bootstrap front-end framework with Django as the backend framework. Django comes with a pre-built feature rich user management solution, which we used to control access to data stored in Elasticsearch. Figure 1 shows a schematic view of the design of GenESysV.

### User interface

Given the widespread use of high throughput sequencing technologies, one goal of this initiative was to make candidate disease-causing variant identification accessible to a wide range of users, including scientists and clinicians. This required GenESysV’s interface to strip away the complexity of building queries using hundreds of annotations.

**Fig. 2:**
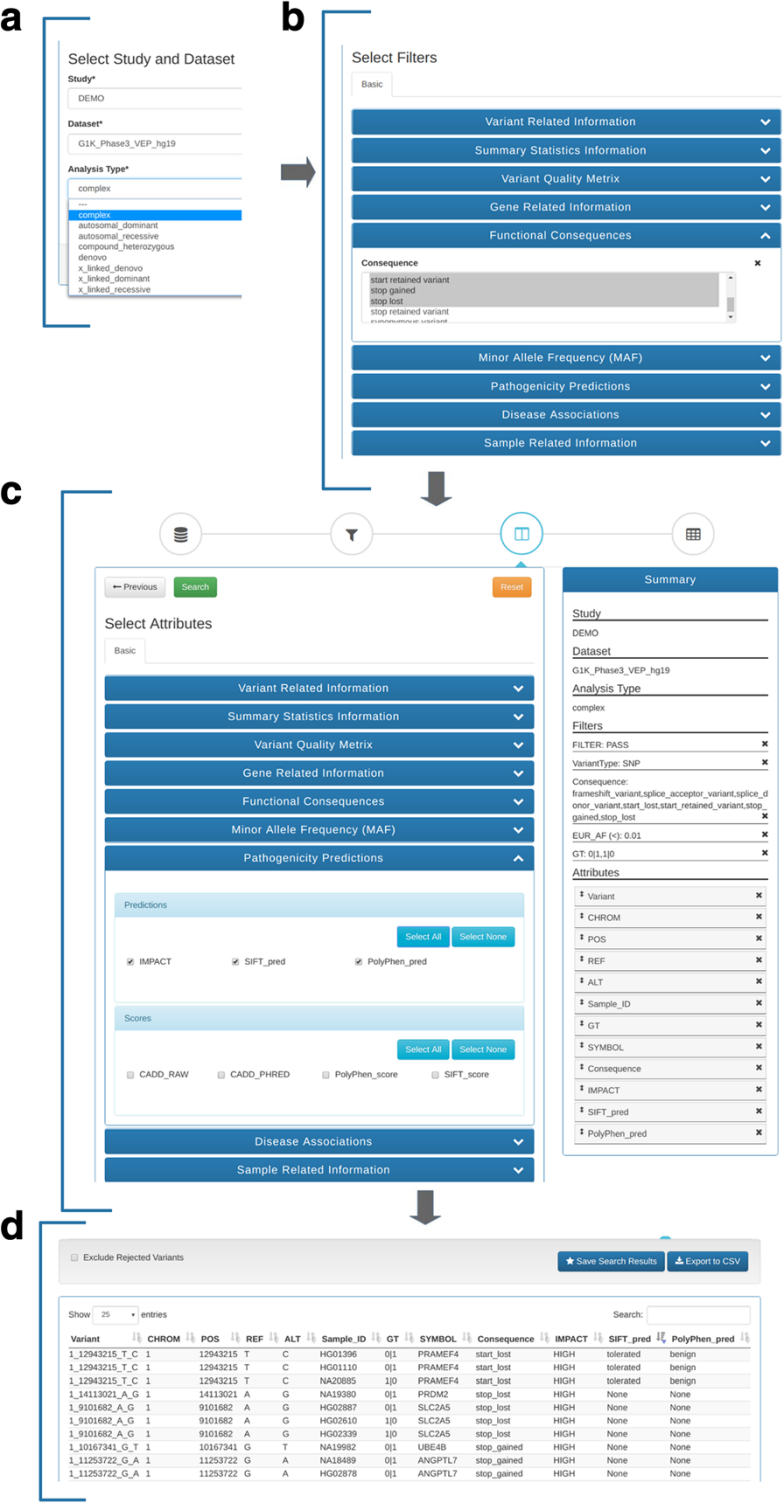
The Interface of GenESysV and querying process. Querying process starts with the selection of study name, dataset name, and analysis type (**a**), followed by setting filters (**b**) and selecting variant/annotation related attributes (**c**). The output is a table displaying up to 400 records (**d**). The full results can be obtained by clicking the “Export to CSV” button (Top right)

We accomplished the above goal in two ways. First, inspired by the GUI design of BioMart [23], we grouped the hundreds of annotations according to their logical relevance – thereby facilitating users to locate them in the filter and attribute sections (Fig. 2) quickly and intuitively. For example, all of the variant related attributes are independent of the sample related fields, therefore putting them into a single “Variant Related Information” category. The sample genotype and read-depth, etc. are specific to each sample and allows them to be grouped into the “Sample Related Information” category. In addition, the data type determines how a given attribute should be used to set up filters. For example, a numeric data type can be used to create range filters to accept threshold values for filtering and a categorical data type can be used to create a dropdown list in the user interface for filter value selection. With this grouping strategy, in conjunction with the use of data types, we designed our data loading script to generate the GUI configuration file after the Elasticsearch index creation. This GUI configuration file guides the automatic creation for both the filter page and attribute page in the web interface. Unlike the “MartConfigurator” tool used in the BioMart system [23], this design simplified user interface creation and eliminated the need to manually edit an XML configuration file, a process that is both tedious and error-prone. Second, we designed GenESysV to guide the user through a three-step wizard for identifying candidate disease-causing variants. As shown in Fig. 2, the first step (Fig. 2a) is for the selection of the study name followed by the selection of the dataset name and analysis type. The various analysis types fall in two major modes, one is for complex diseases or general exploration of the database, and another is for analyzing data for Mendelian inheritance. In the second step (Fig. 2b), users are provided with a filter page that can be used to either include or exclude variants which satisfy the filtering criteria. The third step (Fig. 2c) is for the selection of the attributes which will be displayed in the results table. To help users keep track of the selected filters and attributes, we designed and implemented a panel on the right-hand side of the browser window to display the selected fields, along with the study name, dataset name and analysis type. From this panel, users can review the selected filters and attributes or remove some of them if they are not desired. Users can also re-order the attribute names by dragging-and-dropping, allowing them to customize the ordering of selected attributes on the output page (Fig. 2d) or in the downloaded table. By default, the output window will only display the first 400 records. However, all of the records that satisfy the filtering criteria are available for download if users click the “Export to CSV” button on the tool-bar (Fig. 2d, top right).

### Input file(s)

GenESysV takes annotated VCF files as inputs for data parsing and database (Elasticsearch index) creation. Currently, GenESysV supports VCF files annotated with either of the two commonly used annotation tools: the Ensembl Variant Effect Predictor (VEP) [8] and Annovar [9]. However, GenESysV can parse any existing annotations found in the input VCF before being annotated by the two annotation tools, as long as they are single (as opposed to the “CSQ” from VEP, which contains a list of annotations delimited by commas) attribute-value pairs, separated by semicolons. For single cohort studies of complex diseases, a single VCF file is required. For family-based studies, users need to provide an additional pedigree file. GenESysV also supports studies involving a case and control cohorts. In this case, an additional VCF file from the control cohort is required.

### Parallel processing of input VCF(s) and Elasticsearch index creation

**Fig. 3:**
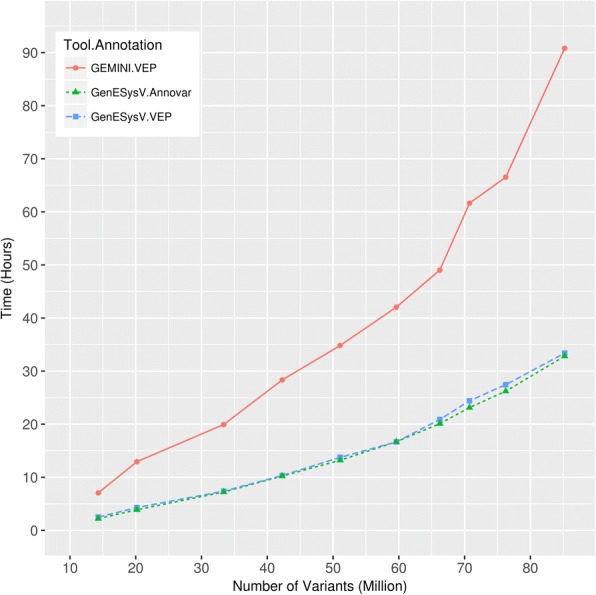
Benchmarking of VCF data import. Comparison of VCF importation between GenESysV and GEMINI. For comparison purposes, data importation performance for Annovar annotated VCF files is also shown in this figure. The phase3 VCF file from the 1000 Genomes Project is downloaded and annotated with VEP or Annovar. A series of VCF files containing the full or subsets of variants is generated by including variants from the first 100, 250, 500, 750, 1000, 1250, 1500, 1750, 2000 samples. These VCF files are used as inputs for importation using the data loading script (load_vcf.py) in our GenESysV package. These tests were performed using a server computer with 24 CPU cores (Intel(R) Xeon(R) CPU E5–2620 v3 @ 2.40GHz) and 128 GB memory (max heap size for Java virtual machine was set to 31 GB using the –Xmx flag, as recommended by the Elasticsearch documentation)

GenESysV is designed for cohort studies with thousands of samples. As data parsing and database index creation are typically slow processes, analyzing large VCF files involving tens of millions of variants from hundreds to thousands of samples can take a substantial amount of time. Thanks to the context independent nature of simple variants (SNVs and INDELs, as opposed to complex structural variants such as translocations), a VCF file for simple variants can be divided into several sections or genomic intervals based on the number of available CPU cores allowing each section to be parsed in parallel by a single CPU core. To this end, we utilized the “grabix” [24] tool developed in the GEMINI project to simultaneously access each of the genomic intervals in the input VCF file. For case and control cohort VCF data parsing, we utilized the “tabix” [25] tool developed in the 1000 Genomes Project [26] for quickly accessing variants in the same genomic intervals between the case and control VCF files. In the database index creation step, we used the “parallel_bulk” tool from the Elasticsearch Python helpers package. This design strategy enabled GenESysV to process VCF files containing hundreds to thousands of samples in a short amount of time (Fig. 3).

### Controlled access for authorized users

GenEsysV was also designed for a multi-investigator environment. Many studies generated genomic variant data that are intended to be accessed by a small group of authorized users. To meet this need, GenESysV was designed and implemented to allow access control by only users with proper access privileges. The database administrator who is involved in the setting up of the software can create user accounts and specify data access privileges. The lab users can then login from their own computers with the provided login credential.

### Supported analysis modes

GenESysV supports analysis for both complex and single gene Mendelian diseases. In the complex disease analysis mode, users can additionally analyze case-control datasets for differential allele distribution between the two cohorts if a VCF file from a control cohort is provided. For family-based studies, supported inheritance patterns include autosomal de novo, autosomal dominant, autosomal recessive, compound heterozygous, as well as X-linked de novo, X-linked dominant and X-linked recessive (See Additional file 1 for rules used in analyzing Mendelian analysis).

### Post query review and curation of variants

In most cases, the called variants are filtered purely by computational methods based on certain types of statistics criteria, such as the Variant Quality Score Recalibration tool in GATK [27], because experimental validation is only feasible for a short list of variants. Due to the limitations of the sequencing technologies, which tends to produce base calls less accurate at the ends of the short-read sequences as well as the limitations of the current generation of alignment software, some of the called variants can be results from alignment artifacts. To examine variants in the search results, users can use external software tools such as IGV [28, 29] to examine the bam files for visualizing the region around the variants and flag them as “Approved” or “Rejected” on the individual variant report page. These flags can be saved and used to remove the “Rejected” variants to further narrow down the most likely disease-causing variants. This helps in creating a clean list of variants for pathway or interaction analysis thus allowing for more meaningful results.

### Save queries for subsequent use

It is often desirable to record which filter terms and associated values/thresholds are used for data extraction as well as which attributes are selected to build the final results. Manually recording each of the filters and attributes is tedious and error-prone, especially for datasets with a large number of filter terms and attributes. To get around this, GenESysV has a tool that saves user queries to their associated account. These saved queries can be conveniently re-run by the same user at a later time without re-selecting/setting the filters and attributes.

### Links to external tools

To get more information from the publicly available resources for variants of interest, GenESysV contains hyperlinks for each of the variants in the variant report page. These hyperlinks can open a UCSC genome browser [30] page to show the location of the variant and its surrounding region, or bring the users to the DECIPHER [31] website to examine clinical information related to variants of interest. To further help users to gain insights into the biological significance of a short list of selected variants and the genes they affect, GenESysV hyperlinks these genes to the GeneMANIA [32] website to perform pathway and interaction analysis.

## Results

### VCF parsing and data loading performance

We compared GenESysV with another well-known system, GEMINI, as it can be easily installed locally and is also capable of handling large whole genome sequencing datasets from multiple samples. To demonstrate the performance of GenESysV’s VCF parsing and database creation, we utilized the full 1000 Genomes Project phase3 dataset [26] as well as a series of VCF files containing a subset of it (See Additional file 1 for details). As shown in Fig. 3, data loading time grew exponentially as the number of variants and samples increases for both GEMINI and GenESysV. However, GenESysV has a much slower rate of increase in loading time as the number of variants/samples increases. Unlike GEMINI, GenESysV can still load VCF files with a number of variants beyond 85 million (the largest available public data at the time this writing) in a reasonable amount of time. In general, GenESysV has a data loading speed approximately two and a half times faster than GEMINI on the same hardware. The full 1000 Genomes Project phase 3 dataset containing 85 (excluding chromosome Y) million variants from 2504 samples can be loaded in less than 34 h using GenESysV under our test system, as compared to nearly 91 h needed by GEMINI (Fig. 3).

Since Annovar is another commonly used annotation tool and the available annotation fields can be as many as three hundred (as opposed to ~ 70 in VEP), we additionally benchmarked data loading using the same set of test files as used above but annotated using Annovar with 35 annotation sources containing 232 annotated features. We found that loading these Annovar annotated VCF files took a similar amount of time as the same set of VCF files annotated with VEP, albeit the Annovar annotated files are two times larger in size due to more annotation fields they contain (Additional file 2: Figure S3).

In addition to testing data loading performance for VCF files annotated with the two most commonly used annotation tools using a 24 CPU core server computer, we further benchmarked data loading with a four and eight CPU core computers, respectively, as many small research groups may not have access to computers with a large number of CPU cores. Results are shown in Additional file 3: Table S1.

To further characterize our loading script’s performance, we also benchmarked VCF data loading under two additional conditions: 1) with a fixed number of variants but an increasing number of samples; 2) with a fixed number of samples but an increasing number of variants. For condition one, the data importing time showed a linear dependency (Additional file 4: Figure S1). However, for condition two, data importing speed showed overall linear dependency but is slower for variants in some genomic intervals (Additional file 5: Figure S2).

One important concern in selecting computational tools is the storage requirement for storing final and intermediate files. When comparing disk-space requirements between GenESysV and GEMINI, we found that GenESysV has a smaller footprint on both the final database size and the temporary storage needed for VCF parsing and importation (Additional file 6: Figure S4).

We lastly profiled memory usage for the two parallelizable processes (i.e. VCF data parsing and Elasticsearch index creation) in GenESysV. We found that the VCF parsing step uses a very small amount of memory, approximately 200 MB/CPU core (Additional file 7: Table S3). For memory usage in Elasticsearch index creation step, since Elasticsearch is Java based and requires a predefined fixed amount of memory to be allocated to the Java Virtual Machine (JVM, with the -Xmx flag) before server startup, we profiled the memory usage inside the JVM. We found that the actual amount of memory used inside JVM is independent of the number of CPU cores used, rather it is proportional to the total amount of memory allocated to the JVM (approximately 80%, Additional file 7: Table S3). We observed that higher number of allocated heap memory does not seem to speed up the data importation process in Elasticsearch once the heap memory is sufficiently large (Additional file 8: Figure S5). Taken together, GenESysV has an overall small memory footprint and is suitable to be deployed on computers with more CPU cores but a limited amount of physical memories.

### Query performance

Fast data loading is certainly advantageous. On the other hand, users will spend more time running queries against the databases. To achieve meaningful and compatible query performance testing, we used similar queries found in the GEMINI paper [18] to test against the datasets from the 1000 Genomes Project phase 3 [26] and a smaller three-sample dataset, the Ashkenazim Trio, generated by the Genome in a Bottle consortium [33]. In all the queries tested, we found better performance in GenESysV as compared to GEMINI for queries executed for the first time (i.e. no caching effect). The same query if executed subsequently generally take less than one second in most cases for both GenESysV and GEMINI (Table 2). Since Elasticsearch can leverage multiple nodes, we also tested if the same query takes less time in a cluster environment. To this end, we imported the full VEP-annotated 1000 Genomes Project phase 3 VCF file into a single computer and a cluster consisting of four nodes each running an instance of Elasticsearch server, respectively. In general, we found the first query takes less time to finish if executed in the cluster environment. However, the execution times for the same query executed subsequently on a single node computer out-performs the cluster setup in general (Additional file 9: Table S2).

**Table 2:** Comparison of query performance between GenESysV and GEMINI^a^

Query	GenESysV filters and attributes	GEMINI query	AshkenazimTrio (6,312,781 variants)		1000 Genomes Project phase3 (2504 samples, 85,211,311 variants)	
			GenESysV	GEMINI	GenESysV	GEMINI
Get all novel and detrimental variants	Filters: Limit Variants to dbSNP_ID: excluded, IMPACT: HIGH, FILTER: PASS.	select chrom, start, ref., alt, qual, impact_severity, filter from variants where in_dbsnp = 0 and impact_severity == ‘HIGH’ and filter is Null	0.73 s/0.21 s	2 m41.35 s/1.06 s	33.22 s/0.49 s	2 m42.80s/0.75 s
			(64)^b^	(74)	(55)	(20)
	Attributes: CHROM, POS, REF, ALT, IMPACT, QUAL, FILTER.					
Get all rare, loss-of-function variants	Filters: EUR_AF (<): 0.01, Consequence: frameshift_variant, splice_acceptor_variant, splice_donor_variant, start_lost, start_retained_variant, stop_gained, stop_lost.	select chrom, start, ref., alt, qual, gene from variants where is_lof = 1 and aaf_1kg_eur < 0.01 and filter is Null limit 400	1.20s/0.34 s	2.39 s/0.35 s	9.97 s/0.53 s	2.60s/0.59 s
			(315)	(269)	(400)^c^	(400)^c^
	FILTER: PASS.					
	Attributes: CHROM, POS, REF, ALT SYMBOL.					
Get rare, loss-of-function variants and is also heterozygous in selected samples	Filters: Consequence: frameshift_variant, splice_acceptor_variant, splice_donor_variant, start_lost, start_retained_variant, stop_gained, stop_lost.	select chrom, start, ref., alt, qual, gene, gts.HG003, gts.HG004 from variants where is_lof = 1 and aaf_1kg_eur < 0.01 and filter is Null" --gt-filter “gt_types.HG003 == HET” or “gt_types.HG004 == HET”	0.71 s/0.37 s	3.21 s/0.47 s	51.47 s/2.28 s	1 m33.57 s/3.52 s
			(239)^e^	(213)	(31)	(36)
	FILTER: PASS, EUR_AF (<): 0.01, Sample_ID: HG003^d^, HG004, GT: 0|1,1|0.					
	Attributes: CHROM, POS, REF, ALT, SYMBOL, Sample_ID, GT.					
Get missense variants in human HLA region	Filters: CHROM: 6, POS (>=): 28477797, POS (<=): 33448354, Consequence: missense_variant.	Select chrom, start, ref., alt, gene, max_aaf_all, impact, rs_ids from variants where chrom = ‘chr6’ and start > = 28,477,797 and end <= 33,448,354 and impact= ‘missense_variant’ limit 400.	0.41 s/0.39 s	3.70s/0.51 s	6.77 s/0.62 s	7.72 s/0.78 s
			(400)^c^	(400)^c^	(400)^c^	(400)^c^
	Attributes: CHROM, POS, dbSNP_ID, REF, ALT SYMBOL, MAX_AF.					

^a^Testing performed in a 16 CPU core (2.3GHz Intel Xeon E312xx (Sandy Bridge, IBRS update)) cloud instance running Ubuntu 16.04 OS, with 32 GB memory and solid state drive. VCF files are annotated with VEP

^b^Query time (No. variants returned). The first number in the query time field is the time spent on the query when the system is cold, i.e. system cache is empty. The second number is the time spent on repeating queries when the data is cached by the first run of the same query. Each query was run three times and the median values are used for reporting

^c^These queries return more than 400 variants (a default upper value set in GenESysV to return for display in the web-browser). To avoid measuring time spent in file downloading, we limited the number of variants returned by GEMINI to 400 to make them compatible

^d^These sample IDs are for the AshkenazimTrio dataset. They are replaced with HG00096 and HG00097, respectively, when testing against the 1000 Genomes Project Phase3 dataset

^e^GenESysV does not always return the same number of variants as GEMINI for the equivalent queries. See supplement material for a possible explanation

## Discussion

For larger genome sequencing projects, the resultant multi-sample VCF file can be tens of gigabytes in the compressed form and will normally take a prohibitively long time to load under most currently available systems. For systems using centralized web-servers, uploading big VCF files is not practical. Another challenge that larger datasets may impose is the slowness of performing queries. In this work, we have benchmarked data importation and query performance for GenESysV and GEMINI. We have demonstrated that GenESysV is advantageous over GEMINI for these two benchmarks. Its intuitive graphical user interface allows a broader range of researchers to perform data mining in a very user-friendly way. It is worth noting that GEMINI performs additional annotations during data importation and also creates compressed genotype data, therefore a direct comparison for data parsing and loading may not be possible. Nevertheless, GenESysV is a time saver for importing large VCF files. We also shown in this work that although VEP annotated VCF file size is much smaller (with fewer annotations) than the same VCF file annotated with Annovar (with more annotations, e.g. 25GB vs. 55 GB for the 1000 Genomes Project phase 3 VCF file annotated with VEP or Annovar, respectively), there is little difference in terms of data parsing and Elasticsearch index creation time. This is most likely due to the fact that VEP annotation (the CSQ field) annotates variants based on the individual transcripts, therefore resulting a “nested structure” (in Elasticsearch term) in the parsed json files required by Elasticsearch. This causes annotations for each of the variants and transcript combos to be treated as a single “document” (in the Elasticsearch term) and therefore contributes to a larger index or database size.

In general, we found that data importing time (parsing and Elasticsearch index creation) is linearly related to the number of samples in a multi-sample VCF file if the number of variants is held constant. However, data importing speed can be slower for variants in some genomic intervals as shown in Additional file 5: Figure S2. The likely reason could be the differing nature of the variants in some chromosomal regions. Some variants in these regions may be more common among the study population and/or are in gene-rich regions (therefore have extensive annotation). Since a VCF file is a two-dimensional data matrix (variants by samples), increasing the number of samples under a study also increases the number of variants. This explains the exponential increase of data importation time when the number of samples (therefore variants) is increasing (Fig. 3).

At the time of this writing, we noticed other database technologies [34, 35] are being developed to specifically handle sparse data like the genomic variants data. These technologies hold promise for reducing disk space requirement and database creation time as well as improving query performance, and may provide alternative options for powering future versions of GenESysV.

## Conclusions

High-throughput sequencing technologies are revolutionizing human genetic research and causal variants discovery. With the broad adoption of these technologies, obtaining a short list of highly probable causative or disease-associated variants in a fast and efficient way becomes a hurdle to most researchers. GenESysV is developed to alleviate this problem and to our knowledge, it is also the first tool to be able to handle genomic variant dataset ranging in size from a few to thousands of samples and still maintain fast data importation and good query performance. It has a very intuitive graphical user interface and can also be used in studies where secured data access is an important concern. We believe this tool will benefit the human disease research community who are utilizing high-throughput sequencing or genotyping technologies to interrogate the genetic variants in the human genome.

## Availability and requirements

**Project name**: GenESysV

**Project homepage**: https://github.com/ubccr/genesysv

**Operating system**: Linux

**Programming language**: Python3

**Other requirements**: Java 1.8.0 or higher, Elasticsearch 6.3.1 or higher

**License**: GLP-2

**Any restrictions to use by non-academics**: license needed

## Additional files

Additional file 1: Supplementary information for Methods and Mendelian inheritance analysis rules. (DOCX 20 kb)

Additional file 2: **Figure S3.** Comparison of data parsing and Elasticsearch index creation times between VEP and Annovar annotated VCF files. Input VCF files are the same as used in Fig. 3. (TIFF 9492 kb)

Additional file 3: **Table S1.** VCF data loading under two different hardware settings. (DOCX 9 kb)

Additional file 4: **Figure S1.** VCF data importing times for input files with an increasing number of samples but a fixed number of variants. Variants on chromosome 1 (6,500,542 variants) from an Annovar annotated 1000 Genomes Project Phase3 VCF file is selected and used to create a series of VCF files containing the first 100, 250, 500, 750, 1000, 1250, 1500, 1750, 2000, 2250 and 2504 samples. These VCF files are used for benchmarking data importation. See Additional file 9 for details. (TIFF 9492 kb)

Additional file 5: **Figure S2.** VCF data importing times for input files with a fixed number of samples but an increasing number of variants. The VEP annotated 1000 Genomes Project Phase3 VCF file is used as input to create a series of VCF files to include the first 10, 20, 30, 40, 50, 60 and 70 million variants. These files (including the full VCF file containing the 85 million variants) are used as inputs for benchmarking data importation. See Additional file 9 for details. (TIFF 9492 kb)

Additional file 6: **Figure S4.** Comparison of disk space usage between GenESysV and GEMINI. (TIFF 9492 kb)

Additional file 7: **Table S3.** VCF data parsing and Elasticsearch index creation under different hardware and system settings. (DOCX 10 kb)

Additional file 8: **Figure S5.** Comparison of Elasticsearch index creation time between different Java Virtual Machine heap sizes. (TIFF 9492 kb)

Additional file 9: **Table S2.** Comparison of query performance between a single computer and a four-node cluster. (DOCX 9 kb)

## Abbreviations

GenESysV: Genome Exploration System for Variants
VCF: Variant Calling Format
VEP: Variant Effect Predictor
WES: Whole Exome Sequencing
WGS: Whole Genome Sequencing
