# Supplementary material for: GenESysV: a fast, intuitive and scalable genome exploration open source tool for variants generated from high-throughput sequencing projects
Source: BMC Bioinformatics. 2019 Jan 31;20:61. doi: 10.1186/s12859-019-2636-5 (PMC6357466; doi:10.1186/s12859-019-2636-5)
Supplement: Supplementary file 1 — Supplementary information for Methods and Mendelian inheritance analysis rules. (DOCX 20 kb) [file 12859_2019_2636_MOESM1_ESM.docx]

# Supplementary information

## Methods

### Comparison of GenESysV and Gemini for VCF data importation performance

We first downloaded phase3 VCF files for individual chromosomes for the 1000 Genomes Project consisting 2504 samples from their ftp site (ftp://ftp.1000genomes.ebi.ac.uk/vol1/ftp/release/20130502/). These VCF files are first run through the vcfbreakmulti tool from the vcflib package to split variant sites with multi alleles, then indels were normalized by using bcftools, followed by annotation with Ensembl Variant Effect Predictor (v92, GRCh37) with parameters of “--everything --dir $VEP_CACHE --dir_plugins $VEP_CACHE/Plugins --vcf --fork 8 --cache --refseq --offline --plugin CADD,$VEP_CACHE/whole_genome_SNVs.tsv.gz,$VEP_CACHE/InDels.tsv.gz --force --cache_version 92”. After annotation, these individual VCF files (excluding VCF file for the Y chromosome) are concatenated using vcf-concat from the vcftools package.

To generate test VCF files with varying number of variants, we created a subset of the VCF file produced above by including the first 100, 250, 500, 750, 1000, 1250, 1500, 1750, 2000 samples. Variants not found in any of the samples in the subset files were removed with a custom Perl script. These VCF files along with the full set of 1KG phase3 VCF file prepared above were used as input for importing into GenESysV or GEMINI database. For importing into GenESysV, we used this command line:

python utils/load_vcf.py –vcf *input.vcf.gz* --tmp_dir tmp –annot vep --hostname localhost --port 9200 --index *test_index_vep* --study_name *BenchMarking* --dataset_name *Test_dataset_vep* --assembly hg19 --num_cores 24. For importing these VCF files into GEMINI, we used this command:

gemini load -v *input.vcf.gz* –t VEP --skip-gerp-bp --skip-cadd --cores 24 *test_gemini.db*. We installed GEMINI v0.20.1 and GenESysV on our CentOS 7 based server with 24 Intel(R) Xeon(R) E5-2620 v3 @ 2.40GHz CPU cores and 128GB memory. Tests for generating data used in Figure 3 were performed on this server.

To benchmark VCF data importing with GenESysV for fixed number of variants but varying number of samples, we used an Openstack cloud instance provided by the University at Buffalo’s Center for Computational Research (CCR). This instance has 16 CPU cores (2.3GHz Intel Xeon E312xx (Sandy Bridge, IBRS update)) and 32GB memory. We used all variants on chromosome 1 (contains 6,500,542 variants) from an Annovar annotated 1000 Genomes Project Phase3 VCF file. A series of VCF files containing the first 100, 250, 500, 750, 1000, 1250, 1500, 1750, 2000, 2250 and 2504 samples are generated from this master VCF file. Each VCF file is used as input for the load_vcf.py script to parse the data and creating Elasticsearch indices. We recorded parsing time and indexing time separately and plotted the results on Additional File 1: Figure S1.

Similarly, to benchmark VCF data importing with GenESysV for datasets with fixed number of samples but varying number of variants, we created a set of VCF files from the VEP annotated full G1K VCF file to include the first 10, 20, 30, 40, 50, 60 and 70 million variants. These files (including the full VCF file containing the 85 million variants) were used as input for the VCF importing script to create Elasticsearch indices. Data parsing and indexing time are recorded and plotted in Additional File 2: Figure S2.

### Comparison of disk space requirements between GenESysV and GEMINI

The VEP annotated phase 3 VCF file from the 1000 Genomes Project was used as input for GenESysV or GEMINI. We recorded the disk usage for both the intermediate files and the final database files. Results were plotted as grouped bar plot as shown in Additional file 4: Figure S4. The “Total Storage Required” is the free disk space need to import this VCF file. Temporary files will be deleted automatically by GEMINI after the final database file is created, or can be deleted either manually or automatically (using the “—cleanup” option under the load_vcf.py command-line) by GenESysV after database creation.

### Comparison of Elasticsearch parallel data bulk loading time between different JVM heap sizes under GenESysV

We used a series of Openstack cloud instances running Ubuntu 16.04 OS. Each instance has a 16 Core CPU (2.3GHz Intel Xeon E312xx (Sandy Bridge, IBRS update)) and 32 (for testing with 4 – 28 GB JVM heap sizes) or 64 (for testing with 32 GB JVM heap size) GB memory. We used the VEP annotated phase 3 VCF from the 1000 Genomes Project as input file and recorded data loading (indexing, exclude parsing) time under each of the JVM heap sizes.

### Comparison for Query performance between GenESysV and GEMINI

**Datasets:** In addition to the 1000 Genomes Project Phase3 data, we also downloaded FASTQ files generated by Genome In A Bottle consortium (GIAB) with the Illumina platform for the AshkenazimTrio from <ftp://ftp-trace.ncbi.nih.gov/giab/ftp/data/AshkenazimTrio> site. These sequence files were used as inputs for an in-house developed BWA and variant calling pipeline (GATK v3.8.0) to produce a VCF file with three samples (containing 6.3 million variants). This VCF file was again normalized and annotated with VEP using methods above and subsequently loaded into GEMINI and GenESysV.

**Testing:** In general, the first query will take much longer time than the same query performed repeatedly as the later queries use information cached in the system. To get the “worst” case query performance, i.e. the time to get a query results when a query is first run, we cleared the system cache for GEMINI and the Elastichsearch cache for GenESysV before running each query. The command we used to clear system cache is “echo 3 > /proc/sys/vm/drop_caches” (under root user account). To clear Elasticsearch cache, we used the command “curl -X POST "localhost:9200/_cache/clear"”. For queries that can result a large number of records, we added the clause “limit to 400” to the GEMINI query to make it return the same number of records as used in GenESysV in order to avoid measuring time spent in file IO.

# Discrepancy in number of variants returned by GenESysV and GEMINI

For the test queries we used, GenESysV and GEMINI returned slightly different number of variants for both the AshkenazimTrio and the 1000 Genomes Project phase3 datasets. For the first query in Table 2, GEMINI returned variants that are also in dbSNP and also failed to return several variants in chrX. We examined the input VCF files and confirmed the correctness of GenESysV. This may be due to the out-of-dated annotation data used in GEMINI during data importation. For test query 3, GEMINI returned a few extra variants which contain missing genotype information in one of the two samples under consideration. These missing genotypes are treated as alternative allele by GEMINI.

### Benchmarking GenESysV for query performance under cluster environment

We created a four-node cluster using CCR’s Openstack cloud platform. The master node has 16 CPU cores (2.3GHz Intel Xeon E312xx (Sandy Bridge, IBRS update)) and 32 GB memory. The other three nodes each has 8 CUP cores and 16 GB memory. All of the four nodes run an instance of Elasticsearch server and configured to form an Elasticsearch server cluster. We imported the full set of the 1000 Genomes Project phase3 VCF (excluding chromosome Y) annotated with VEP using the master node. We also imported the same VCF file into a single cloud instance (16 CPU cores and 32 GB memory) in order to compare query performance under the two different setup. The first three queries used for benchmarking query performance under the 24 CPU core server were used for testing.

## Mendelian inheritance rules used in implementing Mendelian inheritance analysis:

Autosomal dominant rules:

- MUST: GT in ["0/1", "0|1", "1|0"]

- MUST: Phenotype=="2"

- IF Mother_Phenotype=="2", THEN:

- MUST: Mother_Genotype in ["0/1", "0|1", "1|0"]

- MUST: Father_Genotype in ["0/0", "0|0"]

- ELSE IF Father_Phenotype=="2", THEN:

- MUST: Mother_Genotype in ["0/0", "0|0"]

- MUST: Father_Genotype in ["0/1", "0|1", "1|0"]

Autosomal recessive rules:

- Based on annotation:

- VEP

- MUST: CSQ_nested.Consequence in ["frameshift_variant", "splice_acceptor_variant", "splice_donor_variant", "start_lost", "start_retained_variant", "stop_gained", "stop_lost"]

- ANNOVAR

- SHOULD: -- match any of the following:

- ExonicFunc_ensGene in ["frameshift_deletion", "frameshift_insertion", "stopgain", "stoploss"]

- ExonicFunc_refGene in ["frameshift_deletion", "frameshift_insertion", "stopgain", "stoploss"]

- Func_ensGene=="splicing"

- Func_refGene=="splicing"

- MUST: GT in ["1/1", "1|1"]

- MUST: Phenotype=="2"

- MUST: Mother_Genotype in ["0/1", "0|1", "1|0"]

- MUST: Father_Genotype in ["0/1", "0|1", "1|0"]

- MUST: Mother_Phenotype == "1"

- MUST: Father_Phenotype == "1"

*De novo* rules:

- MUST: GT in ["0/1", "0|1", "1|0"]

- MUST: Phenotype=="2"

- MUST: Mother_Genotype in ["0/0", "0|0"]

- MUST: Father_Genotype in ["0/0", "0|0"]

- MUST: Mother_Phenotype == "1"

- MUST: Father_Phenotype == "1"

compound_heterozygous rules:

- FIRST:

- Based on annotation:

- VEP

- MUST: CSQ_nested.Consequence in ["frameshift_variant", "splice_acceptor_variant", "splice_donor_variant", "start_lost", "start_retained_variant", "stop_gained", "stop_lost"]

- ANNOVAR

- SHOULD: -- match any of the following:

- ExonicFunc_ensGene in ["frameshift_deletion", "frameshift_insertion", "stopgain", "stoploss"]

- ExonicFunc_refGene in ["frameshift_deletion", "frameshift_insertion", "stopgain", "stoploss"]

- Func_ensGene=="splicing"

- Func_refGene=="splicing"

- MUST: GT in ["0/1", "0|1", "1|0"]

- MUST: Phenotype=="2"

- MUST: Mother_Phenotype=="1"

- MUST: Father_Phenotype=="1"

- EITHER:

-- Case 1

- MUST: Mother_Genotype in ["0/1", "0|1", "1|0"]

- MUST: Father_Genotype in ["0/0", "0|0"]

-- Case 2

- MUST: Mother_Genotype in ["0/0", "0|0"]

- MUST: Father_Genotype in ["0/1", "0|1", "1|0"]

- THEN:

- For each gene with at least two variants and for each family, phase by transmission, i.e. identify variants pairs that have "0|1" and "1|0" or ("1|0", "0|1" ) genotypes on two different loci.

X-linked dominant rules

- General Rules

- MUST: CHROM=="X"

- Annotation is hg19/GRCh37, exclude the following ranges: [60001, 2699520], [154931044, 155260560]

- Annotation is hg38/GRCh38, exclude the following ranges: [10001, 2781479], [155701383, 156030895]

- Case 1 -- Affected Male

- MUST: Sex=="1"

- MUST: Phenotype=="2"

- MUST: GT in ["0/1", "0|1", "1|0", "1/1", "1|1", "1", "./1", ".|1", "1|." ]

- MUST: Mother_GT in ["0/1", "0|1", "1|0"]

- MUST: Mother_Phenotype=="2"

- MUST: Father_GT in ["0", "0/0", "0|0"]

- MUST: Father_Phenotype == "1"

- Case 2 -- Affected Female

- MUST: Sex=="2"

- MUST: Phenotype=="2"

- MUST: GT in ["0/1", "0|1", "1|0"]

- EITHER:

- Case 1:

- MUST: Mother_GT in ["0/0", "0|0"]

- MUST: Father_GT in ["0/1", "0|1", "1|0", "1", "./1", ".|1", "1|."]

- MUST: Father_Phenotype=="2"

- MUST: Mother_Phenotype == "1"

- Case 2:

- MUST: Mother_GT in ["0/1", "0|1", "1|0"]

- MUST: Father_GT in ["0/0", "0|0", "0"]

- MUST: Mother_Phenotype=="2"

- MUST: Father_Phenotype == "1"

x_linked_recessive rules

- General Rules

- Based on annotation:

- VEP

- MUST: CSQ_nested.Consequence in ["frameshift_variant", "splice_acceptor_variant", "splice_donor_variant", "start_lost", "start_retained_variant", "stop_gained", "stop_lost"]

- ANNOVAR

- SHOULD: -- match any of the following:

- ExonicFunc_ensGene in ["frameshift_deletion", "frameshift_insertion", "stopgain", "stoploss"]

- ExonicFunc_refGene in ["frameshift_deletion", "frameshift_insertion", "stopgain", "stoploss"]

- Func_ensGene=="splicing"

- Func_refGene=="splicing"

- MUST: CHROM=="X"

- Annotation is hg19/GRCh37, exclude the following ranges: [60001, 2699520], [154931044, 155260560]

- Annotation is hg38/GRCh38, exclude the following ranges: [10001, 2781479], [155701383, 156030895]

- Case 1 -- Affected Male

- MUST: Sex=="1"

- MUST: Phenotype=="2"

- MUST NOT: GT in ["0/0", "0|0", "0"]

- MUST: Mother_GT in ["0/1", "0|1", "1|0"]

- MUST: Mother_Phenotype==1

- Case 2 -- Affected Female

- MUST: Sex=="2"

- MUST: Phenotype=="2"

- MUST: GT in ["1|1", "1/1"]

- MUST: Mother_GT in ["0/1", "0|1", "1|0"]

- MUST: Mother_Phenotype=="1"

- MUST: Father_GT in ["0/1", "0|1", "1|0", "1", "./1", ".|1", "1|."]

- MUST: Father_Phenotype=="2"

X-linked *de novo* rules

- General Rules

- MUST: CHROM=="X"

- Annotation is hg19/GRCh37, exclude the following ranges: [60001, 2699520], [154931044, 155260560]

- Annotation is hg38/GRCh38, exclude the following ranges: [10001, 2781479], [155701383, 156030895]

- Case 1 -- Affected Male

- MUST: Sex=="1"

- MUST: Phenotype=="2"

- MUST: GT in ["0/1", "0|1", "1|0", "1/1", "1|1", "1"]

- MUST: Father_GT in ["0/0", "0|0", "0"]

- MUST: Mother_GT in ["0/0", "0|0", "0"]

- MUST: Father_Phenotype=="1"

- MUST: Mother_Phenotype=="1"

- Case 2 -- Affected Female

- MUST: Sex=="2"

- MUST: Phenotype=="2"

- MUST: GT in ["0/1", "0|1", "1|0"]

- MUST: Father_GT in ["0/0", "0|0", "0"]

- MUST: Mother_GT in ["0/0", "0|0", "0"]

- MUST: Father_Phenotype=="1"

- MUST: Mother_Phenotype=="1"

## Format of pedigree file

For Mendelian inheritance analysis, a pedigree file is required. This file should include the following tab-delimited fields (in the order of):

FamilyID, SubjectID, FatherID, MotherID, Sex, Phenotype, age, affected_sibs_id, affected_sibs_sex, affected_sibs_age, unaffected_sibs_id, unaffected_sibs_sex, unaffected_sibs_age. The following values should be used:

For Sex: 1 == Male, 2 == Female; For Phenotype, 1 == Unaffected, 2 == Affected. Missing values can be filled with “-9”. **Note**: the first six fields are required and the remaining are optional. They are used for analysis involving multiple family members. If sibling information is available, they should be delimited by commas if multiple values exist. Below is an example for a simple single family pedigree file:

$ less ../data/vcf/AshkenazimTrio.ped

#Family Subject Father Mother Sex Phenotype age, affected_sibs_id, affected_sibs_sex, affected_sibs_age, unaffected_sibs_id, unaffected_sibs_sex, unaffected_sibs_age

family1 XG104 -9 -9 2 1 -9 -9 -9 -9 -9 -9 -9

family1 XG103 -9 -9 1 1 -9 -9 -9 -9 -9 -9 -9

family1 XG102 XG103 XG104 1 2 -9 -9 -9 -9 -9 -9 -9

family3 XG302 XG303 XG304 1 2 -9 -9 -9 -9 -9 -9 -9
