# Supplementary figures and images for: GenESysV: a fast, intuitive and scalable genome exploration open source tool for variants generated from high-throughput sequencing projects
Source: BMC Bioinformatics. 2019 Jan 31;20:61. doi: 10.1186/s12859-019-2636-5 (PMC6357466; doi:10.1186/s12859-019-2636-5)

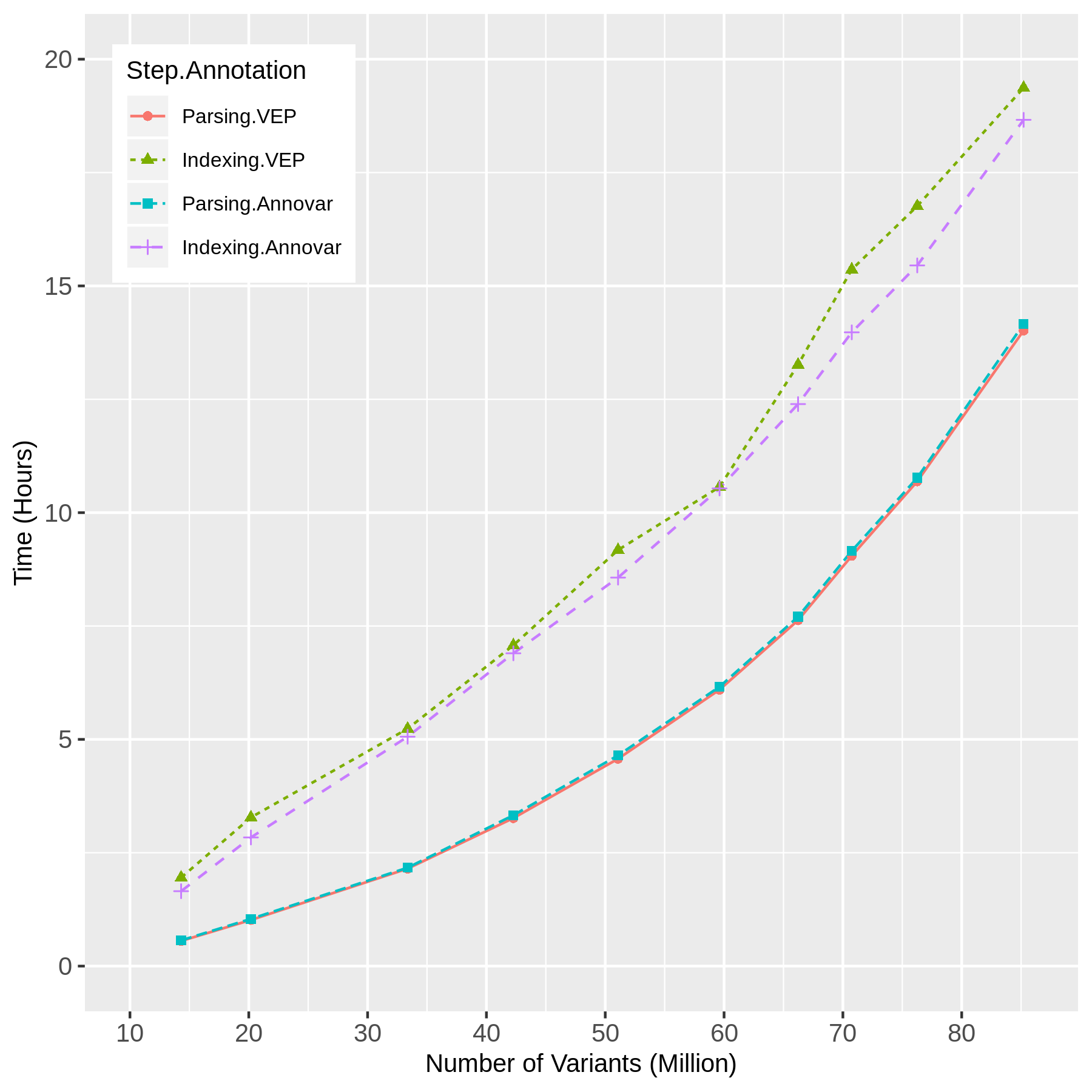

Supplement: Supplementary file 2 — Figure S3. Comparison of data parsing and Elasticsearch index creation times between VEP and Annovar annotated VCF files. Input VCF files are the same as used in Fig. 3. (TIFF 9492 kb) [file 12859_2019_2636_MOESM2_ESM.tiff]

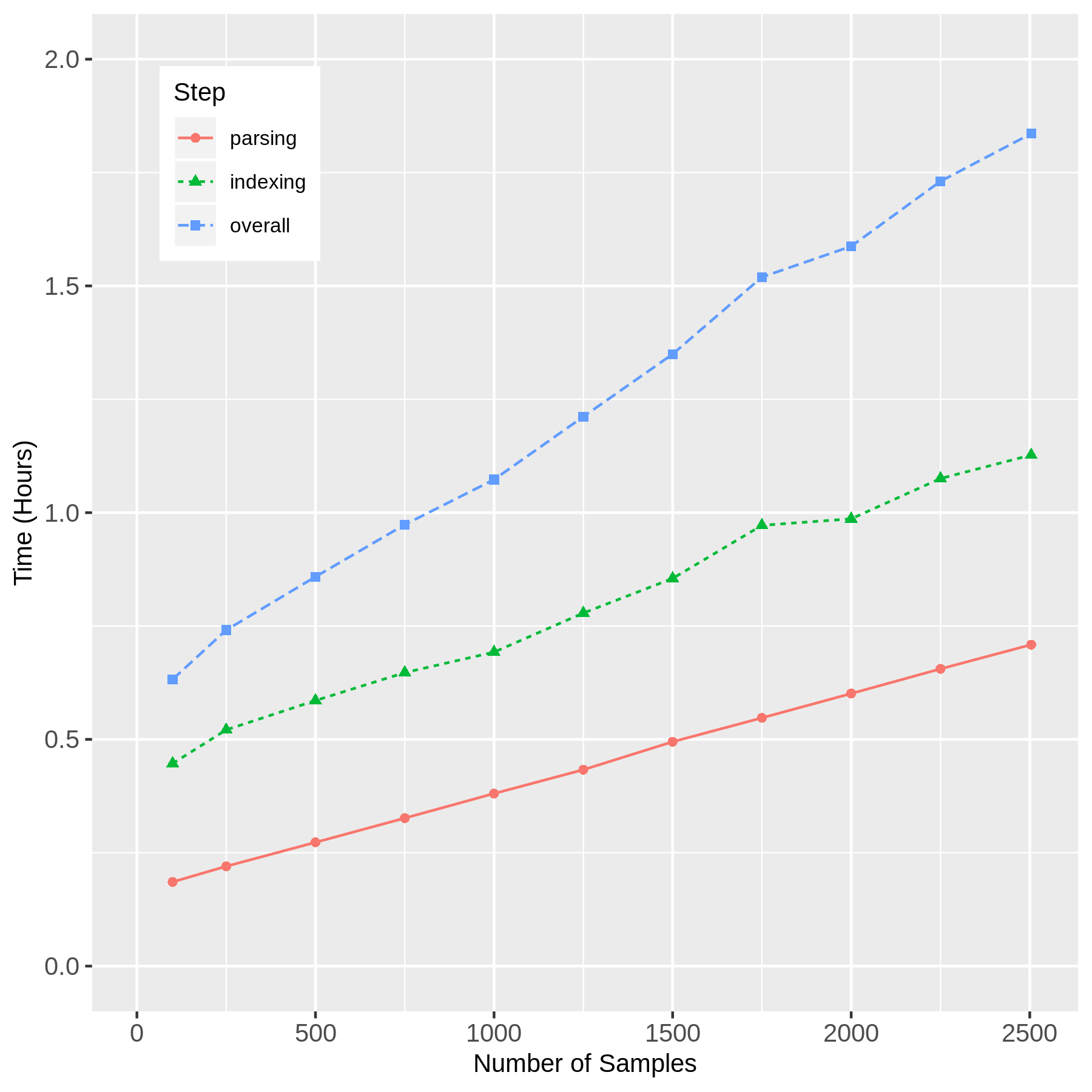

Supplement: Supplementary file 4 — Figure S1. VCF data importing times for input files with an increasing number of samples but a fixed number of variants. Variants on chromosome 1 (6,500,542 variants) from an Annovar annotated 1000 Genomes Project Phase3 VCF file is selected and used to create a series of VCF files containing the first 100, 250, 500, 750, 1000, 1250, 1500, 1750, 2000, 2250 and 2504 samples. These VCF files are used for benchmarking data importation. See Additional file 9 for details. (TIFF 9492 kb) [file 12859_2019_2636_MOESM4_ESM.tiff]

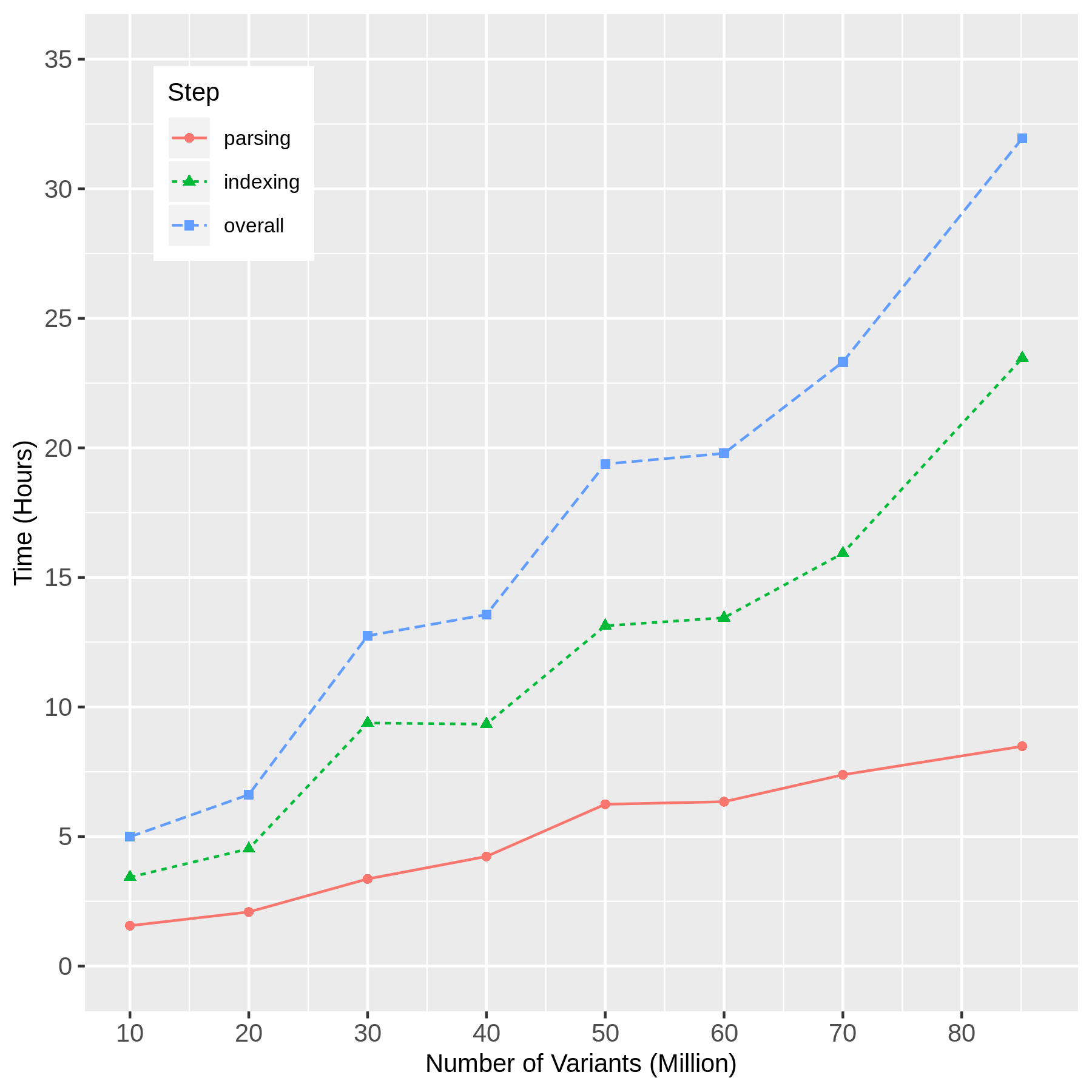

Supplement: Supplementary file 5 — Figure S2. VCF data importing times for input files with a fixed number of samples but an increasing number of variants. The VEP annotated 1000 Genomes Project Phase3 VCF file is used as input to create a series of VCF files to include the first 10, 20, 30, 40, 50, 60 and 70 million variants. These files (including the full VCF file containing the 85 million variants) are used as inputs for benchmarking data importation. See Additional file 9 for details. (TIFF 9492 kb) [file 12859_2019_2636_MOESM5_ESM.tiff]

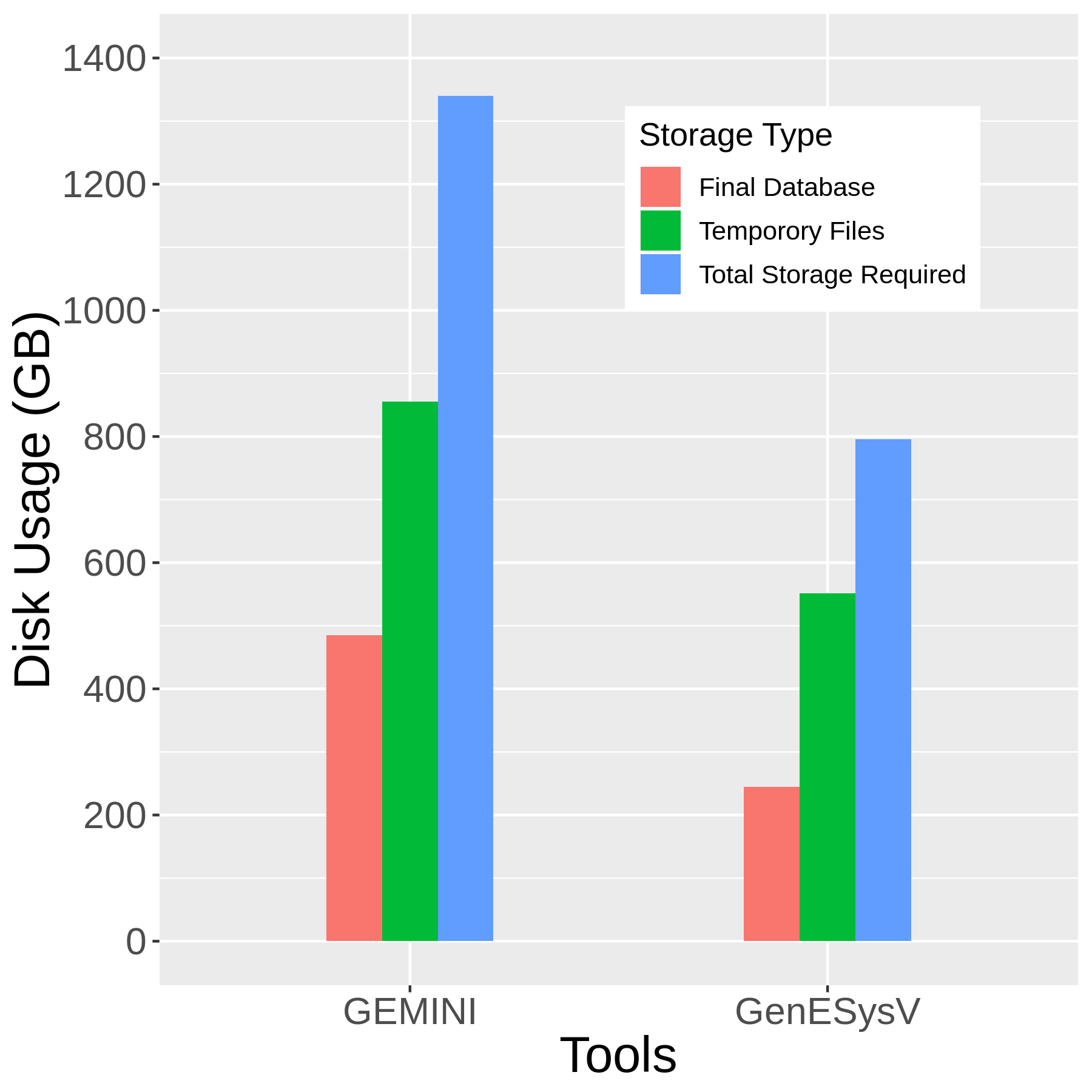

Supplement: Supplementary file 6 — Figure S4. Comparison of disk space usage between GenESysV and GEMINI. (TIFF 9492 kb) [file 12859_2019_2636_MOESM6_ESM.tiff]

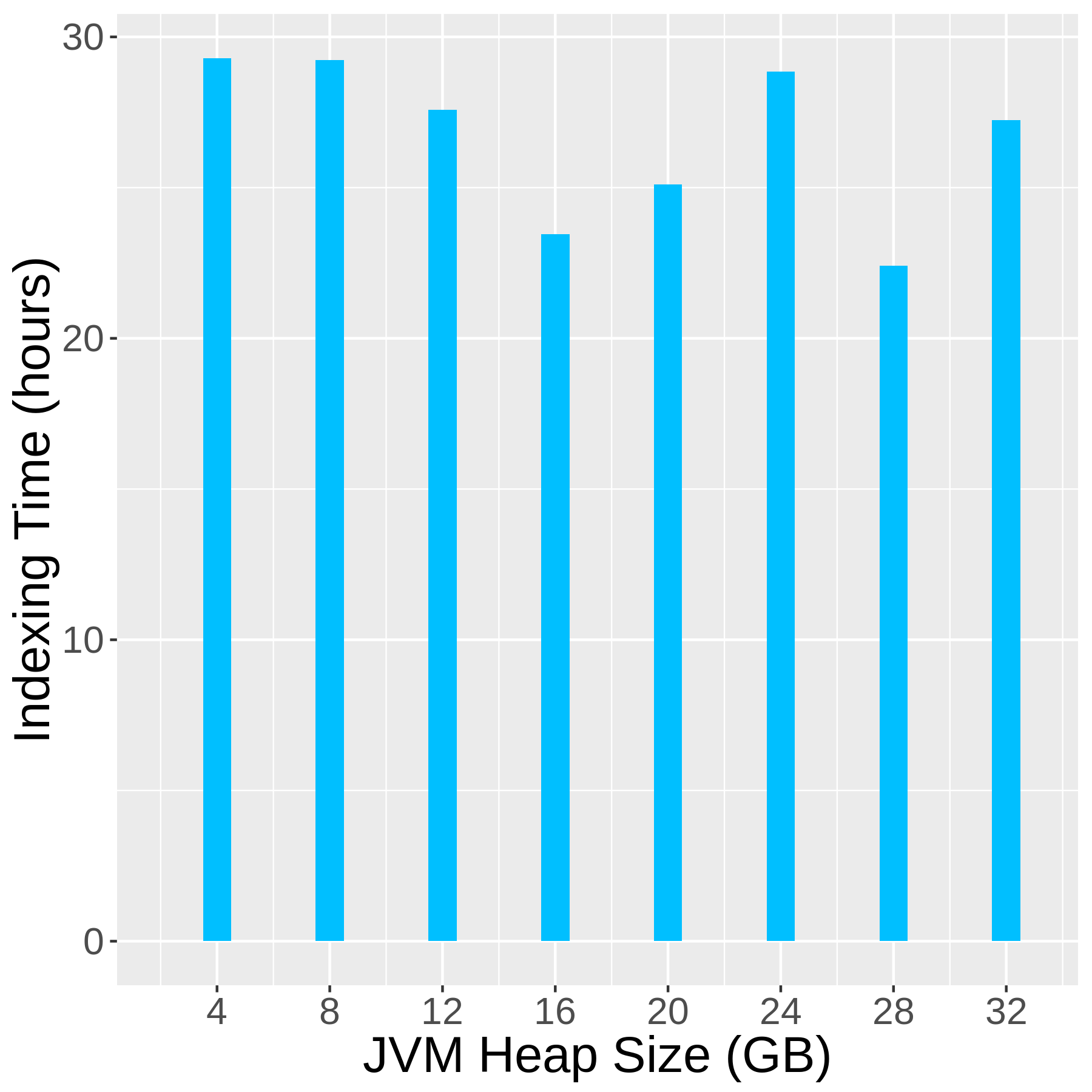

Supplement: Supplementary file 8 — Figure S5. Comparison of Elasticsearch index creation time between different Java Virtual Machine heap sizes. (TIFF 9492 kb) [file 12859_2019_2636_MOESM8_ESM.tiff]
