# Supplementary material for: GenESysV: a fast, intuitive and scalable genome exploration open source tool for variants generated from high-throughput sequencing projects
Source: BMC Bioinformatics. 2019 Jan 31;20:61. doi: 10.1186/s12859-019-2636-5 (PMC6357466; doi:10.1186/s12859-019-2636-5)
Supplement: Supplementary file 3 — Table S1. VCF data loading under two different hardware settings. (DOCX 9 kb) [file 12859_2019_2636_MOESM3_ESM.docx]

**Table S1. VCF data loading under two different hardware settings.** The 1000 Genomes Project Phase3 VCF file and two subsets of this file containing variants from the first 100 and 1000 samples were used for benchmarking the data loading time under four- and eight-CPU core cloud instances. CPU types are Intel Xeon E312xx (Sandy Bridge, IBRS update), 2297.338 MHz with 16384 KB cache.

| Number of Samples (Variants) | Loading Time (Parsing and Indexing, hours) | | | |
| --- | --- | --- | --- | --- |
|  | Four-CPU Cores (16GB RAM, 8GB JVM heap size) | | Eight-CPU Cores (32GB RAM, 16GB JVM heap size) | |
|  | VEP | Annovar | VEP | Annovar |
| 100 (14,304,932) | 4.46 | 4.65 | 3.36 | 3.01 |
| 1000 (51,079,151) | 33.44 | 24.88 | 18.45 | 16.90 |
| 2504 (85,211,311) | 81.95 | 80.27 | 42.05 | 44.33 |
