# Supplementary material for: GenESysV: a fast, intuitive and scalable genome exploration open source tool for variants generated from high-throughput sequencing projects
Source: BMC Bioinformatics. 2019 Jan 31;20:61. doi: 10.1186/s12859-019-2636-5 (PMC6357466; doi:10.1186/s12859-019-2636-5)
Supplement: Supplementary file 7 — Table S3. VCF data parsing and Elasticsearch index creation under different hardware and system settings. (DOCX 10 kb) [file 12859_2019_2636_MOESM7_ESM.docx]

**Table S3.** VCF data parsing and Elasticsearch index creation under different hardware and system settings. The 1000 Genomes Project Phase3 VCF file (87 million variants from 2504 individuals) annotated with VEP was used for testing. Tests were performed using Openstack cloud instances provided by the Center for Computational Research, University at Buffalo. CPU model is Intel Xeon E312xx (Sandy Bridge, IBRS update), 2297.338 MHz with 16384 KB cache.

| Number of CPU Cores | Total System Memory (GB) | Parsing | | Indexing | | JVM Heap Size (GB) | |
| --- | --- | --- | --- | --- | --- | --- | --- |
|  |  | Memory Use (GB)* | Time Spent (hours) | Memory Use(GB)* | Time Spent (hours) | |  |
| 4 | 16 | 0.8 | 31.58 | 9.5 | 22.53 | | 12 |
| 8 | 32 | 1.6 | 15.99 | 19 | 20.92 | | 24 |
| 16 | 32 | 3.2 | 8.91 | 19 | 20.42 | | 24 |
| 16 | 64 | 3.2 | 8.17 | 25 | 19.83 | | 31 |

*Memory use is approximate, as it was estimated from the output of Linux “top” command (for VCF parsing) or jconsol (Java Monitoring & Management Console) plots (for Elasticsearch parallel bulk index creation).
