# Supplementary material for: GenESysV: a fast, intuitive and scalable genome exploration open source tool for variants generated from high-throughput sequencing projects
Source: BMC Bioinformatics. 2019 Jan 31;20:61. doi: 10.1186/s12859-019-2636-5 (PMC6357466; doi:10.1186/s12859-019-2636-5)
Supplement: Supplementary file 9 — Table S2. Comparison of query performance between a single computer and a four-node cluster. (DOCX 9 kb) [file 12859_2019_2636_MOESM9_ESM.docx]

**Table S2. Comparison of query performance between a single computer and a four-node cluster.** The first three queries in Table 2 were performed against the 1000 Genomes Project Phase 3 VCF file annotated with VEP. The first number in columns two and three are the times spent on the first test on the query, i.e. no cache effect. Subsequent numbers are for repeated queries after running other dissimilar queries. Tests were performed using Openstack cloud instances provided by the Center for Computational Research, University at Buffalo. CPU types are Intel Xeon E312xx (Sandy Bridge, IBRS update), 2297.338 MHz with 16384 KB cache.

| Query | Single Computer | Four-node Cluster |
| --- | --- | --- |
| Q1 | 33.22/5.24/0.39/2.04 | 16.04/1.59/1.55/0.40 |
| Q2 | 9.97/0.50/0.43/0.93 | 19.04/5.70/8.33/0.50 |
| Q3 | 51.47/0.55/0.26/0.36 | 20.43/5.72/5.02/8.39 |
